# miR-320 accelerates chronic heart failure with cardiac fibrosis through activation of the IL6/STAT3 axis

**DOI:** 10.18632/aging.203562

**Published:** 2021-09-28

**Authors:** Fang Li, Shan-Shan Li, Hui Chen, Jian-Zhi Zhao, Jie Hao, Jin-Ming Liu, Xiu-Guang Zu, Wei Cui

**Affiliations:** 1 Third Division, Department of Cardiology, The Second Hospital of Hebei Medical University, Shijiazhuang, Hebei 050011, PR China; 2 Department of Biochemistry and Molecular Biology, The Hebei Medical University, Shijiazhuang, Hebei 050011, PR China; 3 Department of Cardiology, The Second Hospital of Hebei Medical University and Hebei Institute of Cardiovascular Research, Shijiazhuang, Hebei 050011, PR China

**Keywords:** miR-320, chronic heart failure, cardiac fibrosis, IL6/STAT3/PTEN axis

## Abstract

Cardiac fibrosis could induce abnormal cardiac function and become a novel target for cardiac hypertrophy and chronic heart failure. MiRNA-320 is a crucial miRNA in cardiovascular disease, but it is poorly understood whether it plays a role in cardiac fibrosis pathogenesis. We aimed to identify the specific underlying mechanism of miR-320 in cardiac fibrosis and hypertrophic pathogenesis. In our study, the GEO datasets revealed that STAT3 was significantly highly expressed in cardiomyocyte lines. MiR-320 activation and STAT3 signaling pathways were statistically significantly connected. Furthermore, miR-320 was highly associated with cardiac fibrosis and hypertrophic disease. Interstitial fibrosis was observed in the mice subjected to TAC surgery, markedly enhanced in miR-320 mimics. Mechanistically, we revealed that miR-320 mimics aggravated the pressure overload and induced cardiac hypertrophy and fibrosis via the IL6/STAT3/PTEN axis. MiR-320 mimics accelerated cardiac hypertrophy and cardiac fibrosis via the IL6/STAT3/PTEN axis. These results suggest that targeting miR-320 may represent a potential therapeutic strategy for cardiac hypertrophy and fibrosis.

## INTRODUCTION

Cardiac hypertrophy is characterized as cardiomyocyte enlargement, fibrosis, cardiac contractile dysfunction, and inflammation, which eventually leads to chronic heart failure [1, 2]. Remarkably, it has been shown that abnormal metabolism induced by severe cardiac fibrosis results in cardiac hypertrophy [3–5]. Cardiac fibrosis is a fundamental process characterized by the accumulation deposition of extracellular matrix (ECM), which leads to contractile dysfunction in various heart diseases and is considered an essential contributor to heart failure (HF). Therefore, cardiac fibrosis is highly associated with heart failure and has become a novel target for HF. JAK/STAT signaling is known to promote HF development by facilitating vascular inflammation. STAT3, as a member of the JAK-STAT signaling pathway, is involved in various physiological changes such as cell proliferation and migration. Emerging evidence indicates that STAT3 plays an essential role in developing cardiac fibrosis and hypertrophy [2, 6].

MicroRNAs (miRNAs) are a massive family of small non-protein-coding RNAs implicated in the occurrence and progression of many cardiovascular diseases, including cardiac fibrosis and hypertrophy [7–11]. Among these miRNAs, a miRNA named MicroRNA-320 attracted our attention. MicroRNA-320 plays critical roles in various kinds of cardiovascular and cerebrovascular diseases, including Cardiac Ischemia/Reperfusion, cerebral Infarction, and diabetes with Myocardial Disease. Ren X P et al. indicated that knockdown of endogenous miR-320 protects against I/R-induced cardiomyocyte death and apoptosis [12–14]. However, it has not been investigated whether miR-320 plays a role in myocardial hypertrophy, especially with cardiac fibrosis. Therefore, we assessed whether miR-320 is involved in cardiac fibrosis’s pathophysiology and the molecular mechanism of miR-320 underlying STAT3 on cardiac hypertrophy and fibrosis using human serum samples and hypertrophic mice models.

## MATERIALS AND METHODS

### Bioinformatics

The GSE124176 and GSE104150 datasets were downloaded from the GEO database (https://www.ncbi.nlm.nih.gov/geo/). The differential analysis between the normal group and the case group was operated by the "edgeR" package (|logFC|>2, padj < 0.05) to obtain the differentially expressed mRNAs. GSEA software was conducted to pathway enrichment analysis of target miRNA to study the target miRNA’s mechanism and its target gene.

### Establishment of cardiac hypertrophy models in mice

Male C57BL/6 mice aged between 8–10 weeks were anesthetized by intraperitoneal injection of 3% chloral hydrate. Arterial puncture (22G) was used to intubate the outer sheath, and the ventilator was used to construct control respiration (frequency = 90–100 breaths/min, tidal volume = 0.4–0.5 ml). The mice were placed on the operating table in the supine position and operated under a binocular stereomicroscope. The mice were fed in mouse IVC independent ventilation cage system and the relevant parameters: ventilation times: 15–50 times/h; Air velocity: 0.05–0.18 m/s; Air cleanliness: Class 100; Number of bacteria falling: 0/dish; Noise: ≤ 55dB. The hearts were dissected from the sternal fossa to the second rib level, and the aortic arch was exposed. A 6–0 silk thread was inserted between the cephalic-brachial trunk and the left common carotid artery, ligated with 25G or 27G needles. After cautiously pulling the needle out, we detected the increased volatility of the proximal aortic arch and elevated heartbeats in the mice. The layer was sutured, and the incision was closed to the chest. After the successful modeling of TAC operation, 3 groups of mice, model-group, miR-320 OE-group and miR-320 OE + BP-1-102-group were injected with negative control (NC) lentivirus, miR-320 (overexpression(OE)-lentivirus, miR-320 OE-lentivirus + BP-1-102 into the tail vein.

### Echocardiography

Ventricular function was evaluated by Doppler echocardiography (MyLabAlpha, Esaote S.P.A) with a 2.5, 2.5, and 3.75 MHz phased array ultrasonic probe. Cardiac hypertrophy was assessed by measuring both systole and diastole, which obtained the following measurements left ventricular posterior wall thickness in diastole (LVPWD), left ventricular posterior wall thickness in systole (LVPWS), left ventricular internal diameter in diastole (LVIDD), left ventricular internal diameter in systole (LVIDS), left ventricular end-diastolic anterior wall thickness (LVAWD), and ejection fraction (E.F.).

### Histological analysis and Masson’s collagen staining

Five weeks after the TAC operation, the mice were anesthetized with chloral hydrate and fixed on the examination platform. We opened the mice’s thoracic cavities under sterile conditions, perfused them with normal ice saline under normal pressure until the livers turned white, separated the hearts, and froze the apical tissue at −80°C. Then, we fixed the remaining myocardial tissue with paraformaldehyde and dehydrated it with gradient ethanol. After embedding these tissues in paraffin, we soaked them in a 15% sucrose solution in PBS for 18 h at 4°C for brazening. They sliced these tissues into five-μm pieces to prepare for Masson staining and immunofluorescence staining.

### Immunofluorescence staining

C.M.s were fixed with 4% formaldehyde in PBS (pH 7.2) for 25 min and permeabilized in 0.1% Triton X-100 for 10 min, then washed three times in C.M.s with PBS and incubated with a primary antibody (ABCOM, dilution: 1:100) at 4°C overnight. After the secondary antibody, C.M.s have washed with PBS once again and incubated with the nuclear stain DAPI for 15 min. Imaging was completed under an Image-Pro Plus 6.0.

### Luciferase reporter assay

Mice cardiac fibroblasts were co-transfected using commercial Lipofectamine^®^ 2000 following the manufacturer’s instructions. miR-320 mimics or inhibitors. The following oligonucleotide sequences were used: scramble NC, 5′-UUCUC CGAAC GU- GUC ACGUU U-3′; miR-320 mimic, 5′-AAAAG CUGGG UUGAG AGGGC GA-3′, miR-320 inhibitors: 5′-UCGCCCUCU CAACCCAGCUUUU-′3 (Shanghai GenePharma Co., Ltd., Shanghai, China). After 24 h transfection, cells were analyzed by using the Dual-Luciferase Reporter Assay System (Promega).

### Cell viability assay

Mice cardiac fibroblasts cells were seeded in a 96-well plate with 5000 cells/well, and treated with the following conditions: fresh culture medium alone (control), fresh culture medium with different concentrations (0–200 μg/mL) of APS (Sigma-Aldrich), and/or fresh culture medium with 10 μM LPS. Cell viability was assessed by a Cell Counting Kit-8 (CCK-8; Beyotime Biotechnology, Nanjing, China) according to manufacturer’s instructions. Briefly, after treatment, the CCK-8 solution was added to the culture medium and incubated at 37°C for 1 h. The absorbance was read at 450 nm with a microplate reader. Cell viability was calculated by (experimental group absorbance value/control group absorbance value) × 100%.

### Real-time PCR analysis

Total cellular RNA was obtained from mice cardiac tissues or cultured C.M.s by TRIzol (Invitrogen) following the manufacturer protocol, preventing remaining genomic DNA using RNase free DNase-I (Qiagen) following the manufacturer protocol. The quality of RNA was further detected by the ultraviolet spectrometer and formaldehyde-agarose gel electrophoresis. Purified RNA was used for quantitative PCR of miR-320 using MicroRNA-210 Assays (Life Technologies) according to the manufacturer’s commended procedure. Quantitative RT-PCR analysis was performed by Bio-Rad real-time PCR detection system using SsoFast Eva-Green Supermix (Bio-Rad, Hercules, USA). The comparative Ct method was performed to assess the expression of miR-320. Additionally, the PCR products were observed using 1% (wt./vol) agarose gel electrophoresis.

### Western blot analysis

The cardiac tissues and C.M.s were homogenized in a lysis buffer containing 2% Triton X-100. Protein concentration was normalized and determined with a BCA protein assay kit (Thermo Scientific, Rockford, IL). Then separated protein using 10% sodium dodecyl sulfate-polyacrylamide gel electrophoresis (SDS-PAGE) and transferred to a nitrocellulose membrane (G.E. Healthcare, Buckinghamshire, U.K.) and incubated the membranes with the primary antibody (1:1000 dilution, 4°C) overnight and secondary antibodies (1:10000 dilution, room temperature) for 1h. The expression levels were identified by luminous-image analyzer densitometry (Luminescent Image Analyzer lAS-1000: Fujifilm, Tokyo, Japan).

### Statistical analysis

All values were presented as the mean ± SEM. Statistical analysis was performed by SPSS version 20.0. Student *t*-test compared the comparison of two means. ANOVA performed a comparison of more than two means. *P* < 0.05 was considered statistically significant.

## RESULTS

### The association between miR-320 and STAT3

**Figure 1:**
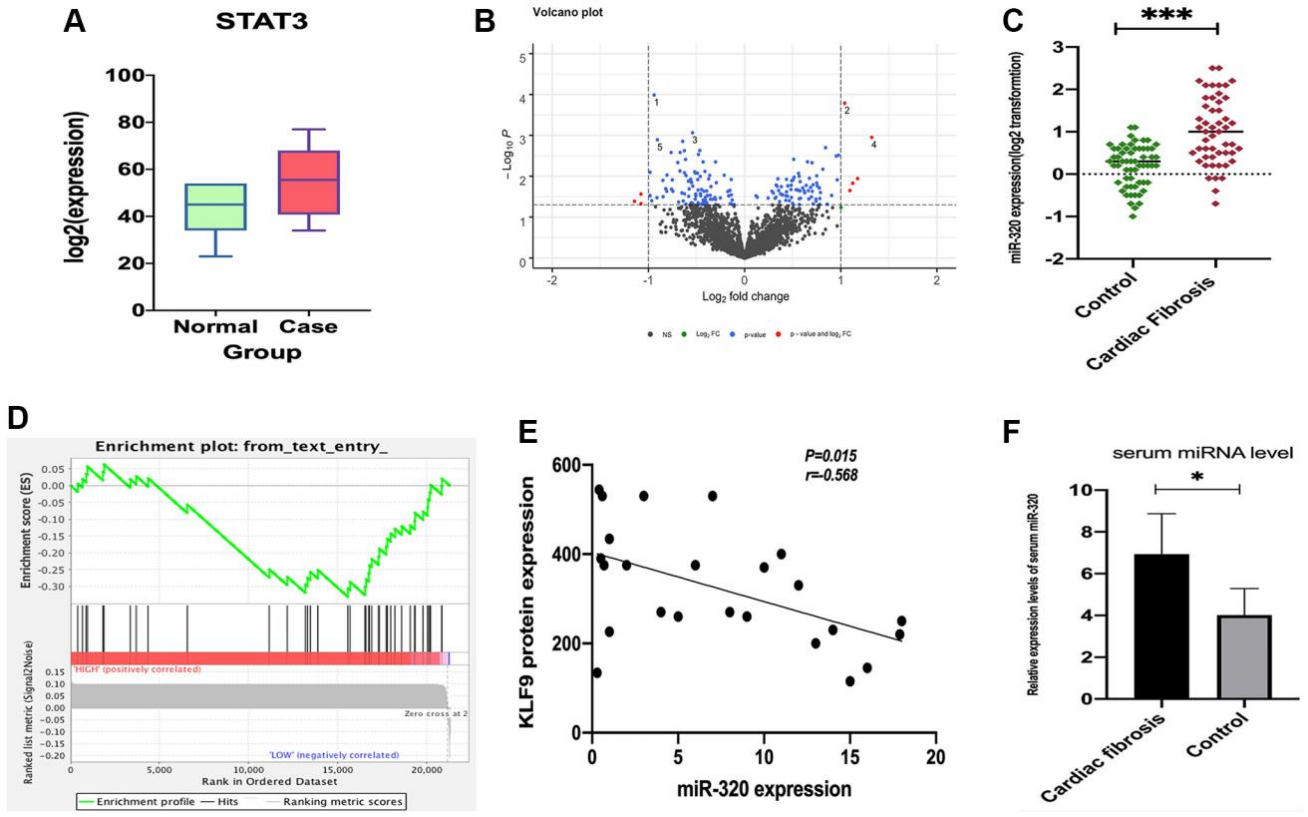
**The expression of STAT3 in cardiomyocyte lines and miR-320 targeted-regulated STAT3.** (**A**) The expression of STAT3 was low in standard samples (green) and high in case samples (red); *P* < 0.05. (**B**) Different expressed miRNA volcano map of standard group and case group in GEO dataset; (**C**) Venn diagram of predicted upstream, down-regulated, differently expressed miRNA for STAT3; (**D**) A heat map depicting miR-320 was higher in the cardiac fibrosis group, as compared with the controls; (**D**) GSEA pathway enrichment analysis results of STAT3; (**E**) Correlation analysis found that miR-320 was negatively correlated with KLF9 protein expression in cardiac tissues. (**F**) The relative expression levels of serum miR-320 were significantly elevated in patients with cardiac fibrosis. *P* < 0.05.

To assess miR-320 expression in cardiac fibrosis patients, we obtained the GSE157331 and GSE104150 datasets from the GEO database, which comprised of cardiac fibrosis samples, and low clinical samples data were ignored. According to the data in GSE157331, we found STAT3 was significantly expressed in cardiomyocyte lines (Figure 1A), and 178 differentially expressed genes were obtained by “edgeR” for differential analysis (Figure 1B). Then we assessed the expression level of mir-320 in cardiac fibrosis from the data in GSE104150. Results revealed that the expression levels of miR-320 were higher in the cardiac fibrosis group, as compared with the controls (Figure 1C). Specifically, KLF9, a member of the Krüppel-like factor (KLF) family, regulates cell proliferation, differentiation, and apoptosis [15]. Additionally, a recent study has highlighted that KLF9 could repress JAK/STAT3 signaling pathway. Most importantly, our gene set enrichment analysis (GSEA) revealed that miR-320 was implicated in regulating the STAT3 signaling pathway, and they were positively related (Figure 1D). Correlation analysis found that miR-320 was negatively correlated with KLF9 protein expression in cardiac tissues (Figure 1E). These results hypothesize that miR-320 is positively correlated with JAK/STAT3 signaling pathway. To further evaluate the role of miR-320 in cardiac fibrosis, we evaluated the level of miR-320 in the serum samples from patients with cardiac fibrosis and matched controls. The relative expression levels of serum miR-320 were significantly elevated in patients with cardiac fibrosis (Figure 1F, *P* < 0.05). Therefore, these results imply that the STAT3 signal was activated and positively associated with miR-320 in cardiac fibrosis and hypertrophic disease. As such, we hypothesize that the regulation of miR-320 may be through the STAT3 signaling pathway in cardiac fibrosis and hypertrophic disease.

### MiR-320 mimics induced left ventricular (LV) hypertrophy and cardiac fibrosis

**Figure 2:**
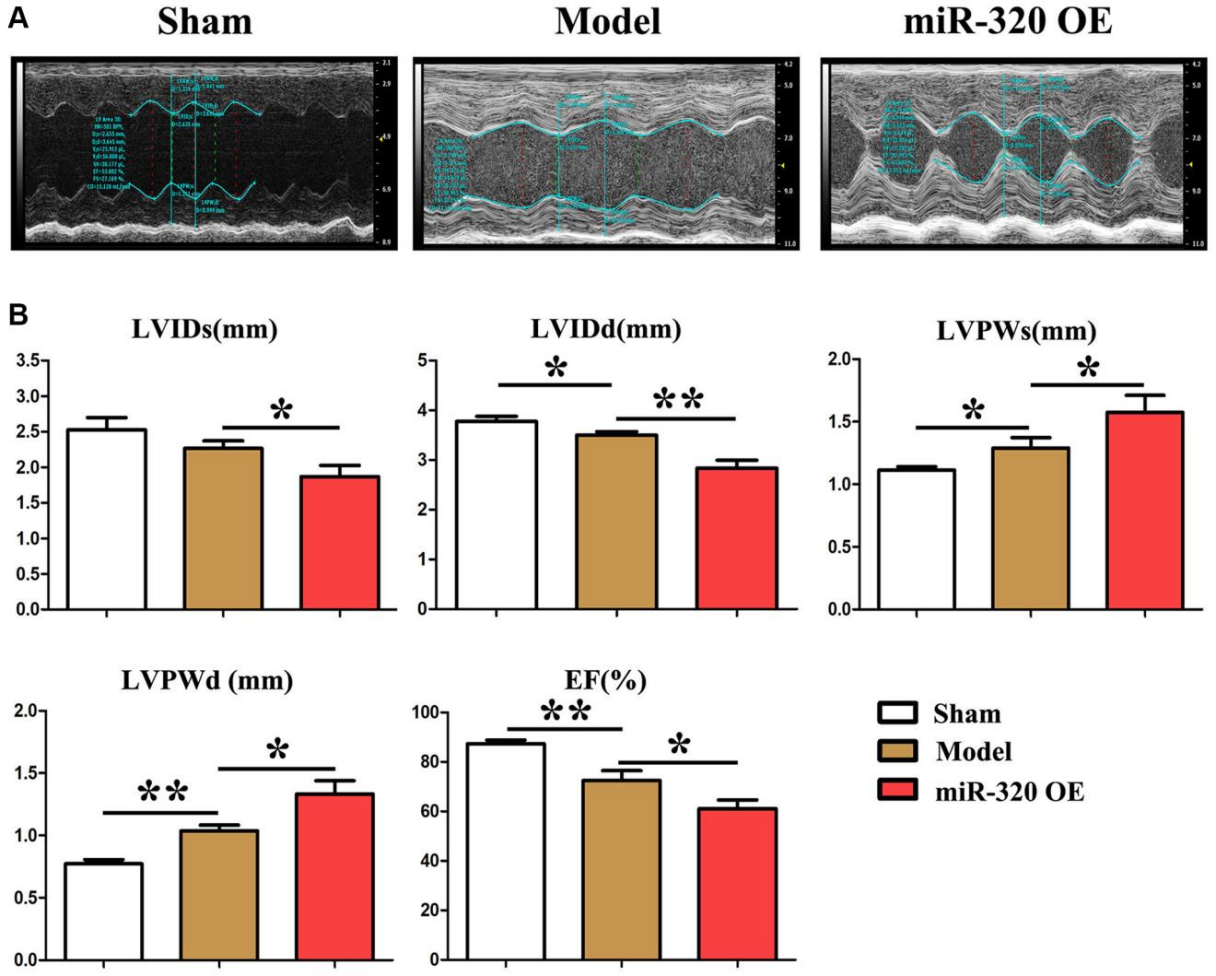
**MiR-320 OE induced left ventricular (LV) hypertrophy and cardiac fibrosis.** (**A**) Representative serial M-mode echocardiography in conscious sham and miR-320 OE and model mice measured before and after TAC; (**B**) Summary data of left ventricular internal dimension diastole (LVIDd), left ventricular internal dimension systole (LVIDs), left ventricular posterior wall dimensions diastole (LVPWd), left ventricular posterior wall dimensions systole (LVPWDs), and ejection fraction (E.F.). *P* < 0.05; miR-320 OE vs. model and sham vs. model.

To investigate the role of miR-320 in fibrosis and hypertrophic disease, miR-320 (overexpression of miR-320) OE, model mice were subjected to pressure overload by transverse aortic constriction (TAC). There was no hypertrophic phenotypic in cardiac function in the sham by echocardiographic scans (Figure 2A). However, after TAC, miR-320 OE mice exhibited severe left ventricle dilation, regional contractile dysfunction (Figure 2A). Cardiac dysfunction arose in model mice and miR-320 OE group after TAC, which was worse in miR-320 OE mice (Figure 2A). Moreover, comparing with the sham and model, miR-320 OE mice exhibited a significant cardiac hypertrophic response, as demonstrated by enlarged LVPWs and LVPWd, reduced LVIDs, LVIDd, and E.F. (Figure 2B, *P* < 0.05). These results suggested that our mice models of cardiac hypertrophy were established successfully and that miR-320 OE could induce worse left ventricular (LV) hypertrophy and cardiac function damage.

### MiR-320 OE was shown to have accelerated the cardiac function damage with significant cardiac fibrosis

**Figure 3:**
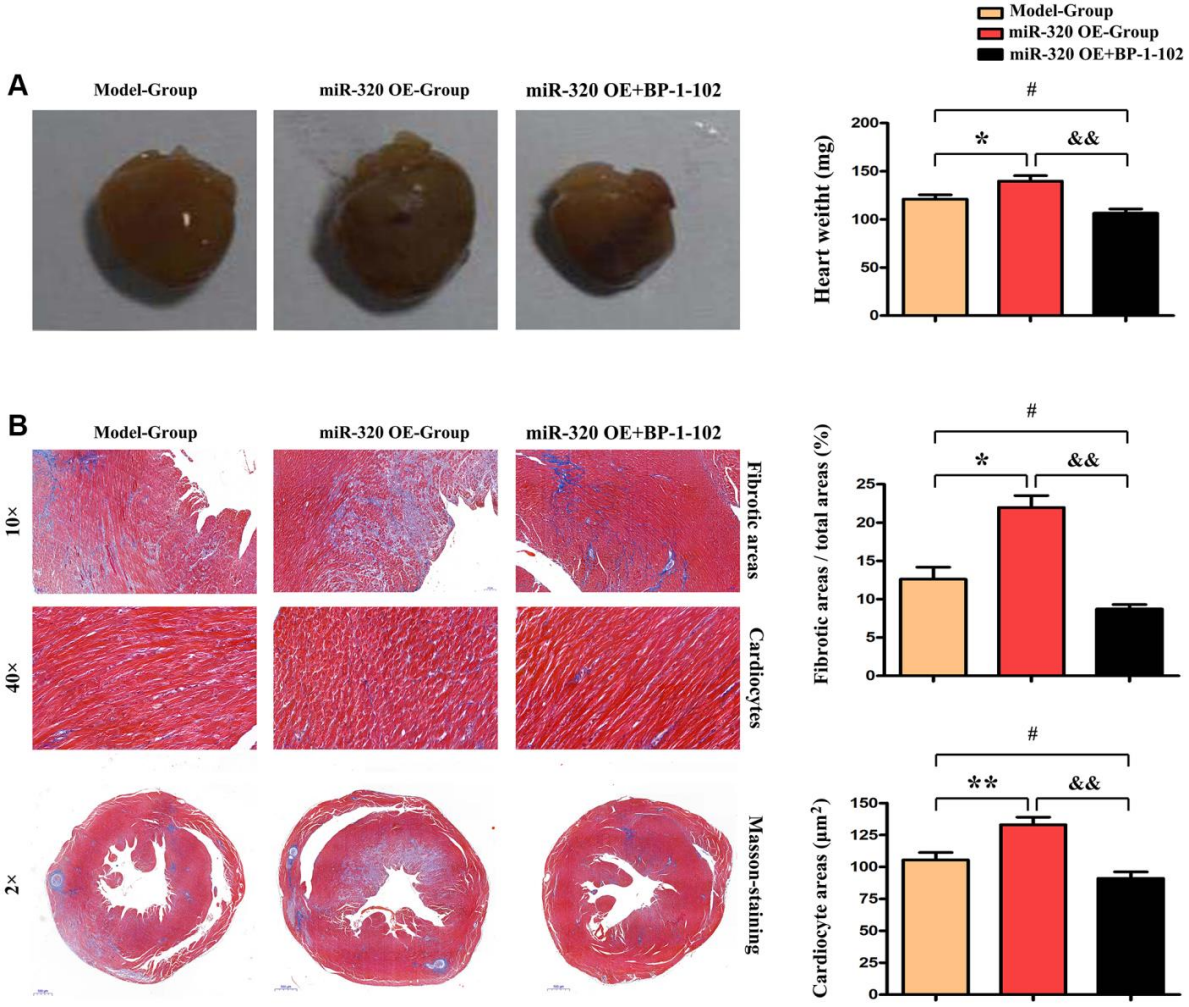
**MiR-320 OE accelerated cardiac function damage with significant cardiac fibrosis.** (**A**) The histological assessment was performed to evaluate cardiac morphology between the model, miR-320 OE, and miR-320 OE + BP-1–102 samples. (**B**) Representative pictures of myocardial connective tissue Masson staining demonstrate cardiac fibrosis and fibrosis area. The quantitative analysis of fibrosis area/total areas, cardiocyte areas, and heart/weight ratio. *P* < 0.05.

Given that cardiac fibrosis is associated with cardiac hypertrophy and chronic heart failure [16], we performed Masson staining *in vivo* to investigate the cardiac fibrosis level in mice’s left ventricular tissues. The histological assessment exposed the differences in cardiac morphology between the model, miR-320 OE, and miR-320 OE + BP-1-102 samples (Figure 3A). Furthermore, severe myocardial interstitial and perivascular fibrosis were found in the heart of miR-320 OE mice after TAC but not in model hearts (Figure 3B). There is, increased myocardial interstitial and perivascular fibrosis by miR-320 OE were significantly reduced in miR-320 OE + BP-1-102 group. The quantitative analysis of fibrosis area/total areas, cardiocyte areas, and heart/weight ratio further showed that cardiac fibrosis was significantly enhanced in miR-320 OE and decreased in miR-320 OE + BP-1-102 group compared with the model group. (Figure 3A and 3B, *P* < 0.05). These results implied that miR-320 OE accelerated cardiac function damage and hypertrophy with a significant cardiac fibrosis increase.

### MiR-320 OE accelerated cardiac fibrosis by increasing the synthesis of collagenous fibers

**Figure 4:**
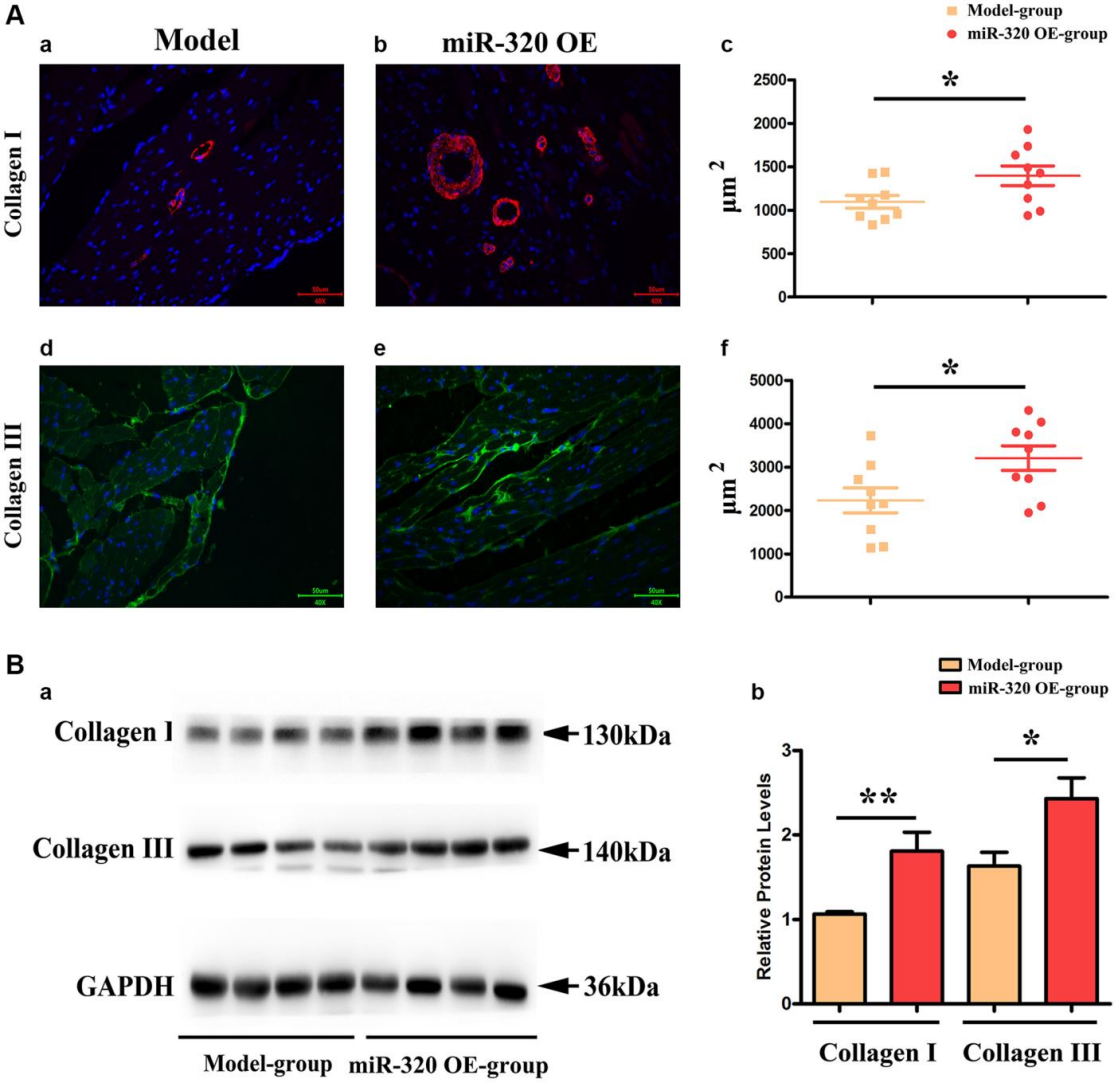
MiR-320 OE increased the type I and III collagen synthesis; (**Aa**–**d**) Representative pictures of type I and type III collagen staining in the left ventricular tissues; (**Ac**–**f**) The expression levels of type I and type III collagen synthesis; *P* < 0.05; miR-320 OE vs. model; (**Ba**) Representative Western blots in the left ventricular tissues to show the collagenous fibers synthesis; (**Bb**) The quantitative analysis of type I and type III collagen; *P* < 0.05; miR-320 OE vs. model.

Given that the accumulation of type I and type III collagen contributes to a dramatic increase in collagenous fibers, which increase cardiac fibrosis and destroy cardiac function [17, 18]. To investigate the effect of overexpression of miR-320 in collagen content, we applied immunofluorescence staining to mice’s left ventricular tissues. The staining identified increased type I and III collagen in the left ventricular tissues after TAC, which was more pronounced in the hearts of miR-320 OE mice (Figure 4Aa–d). Furthermore, we found that the overexpression of miR-320 increased type I and III collagen synthesis, suggesting a deterioration in cardiac fibrosis development. (Figure 4Ac–f, *P* < 0.05).

Moreover, our western blots further confirmed that miR-320 OE significantly enhanced type I and III collagen (Figure 4Ba). The quantitative analysis of relative protein levels further confirmed that miR-320 OE increased type I and III collagen synthesis compared to the model group. (Figure 4Bb, *P* < 0.05) These results implied that miR-320 OE accelerated cardiac fibrosis by increasing the collagenous fibers synthesis.

### MiR-320 OE heightened the TGF-β, IL-6, p-STAT3 signaling and suppressed PTEN *in vivo*

**Figure 5:**
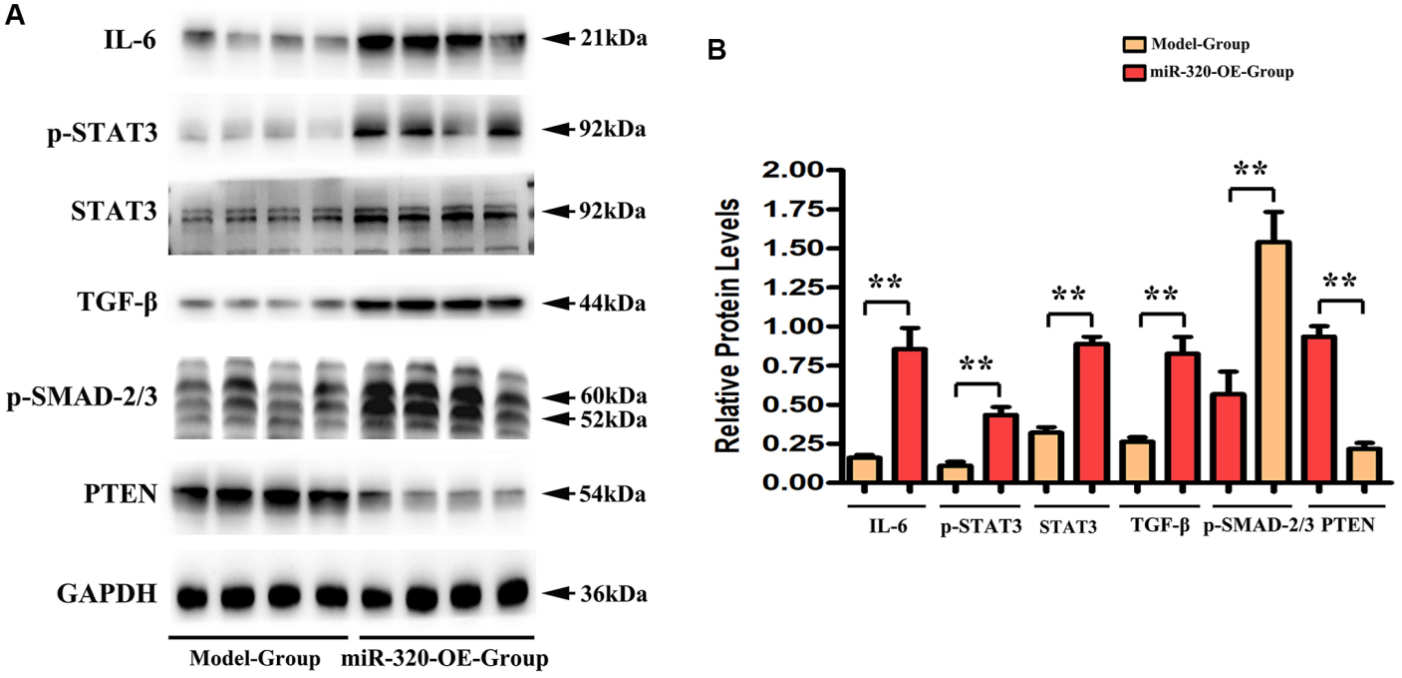
**MiR-320 OE heightened the TGF-β, IL-6, t-STAT3, and p-STAT3 signaling and suppressed PTEN.** (**A**) Representative Western blots in the left ventricular tissues; (**B**) The quantitative analysis of TGF-β, IL-6, p-STAT3, t-STAT3 and PTEN; *P* < 0.05; miR-320 OE vs. model.

Earlier works have indicated that IL-6/STAT3 signaling pathways participate in the elastin breakage and collagenous fibers synthesis. It underlies cardiac hypertrophy and fibrosis progression [19, 20]. Additionally, various studies have confirmed that transforming growth factor-β (TGF-β) is a growth factor that emerged within damaged tissues, where it stimulates fibroblasts and contributes to collagenous fibers synthesis [21, 22]. Remarkably, it has been shown that PTEN negatively correlates to STAT3 signaling activation [23, 24]. To investigate the effect of miR-320 on IL-6/STAT3/PTEN and TGF-β expression, we applied western blots to the left ventricular tissues of mice from both groups. In this study, we found that the expression levels of IL6, TGF-β, p- STAT3, and t-STAT3 were enhanced in the left ventricular tissues of miR-320 OE mice, whereas PTEN was reduced in the miR-320 OE group. (Figure 5A) Our quantitative analysis of relative protein levels further confirmed that IL6, TGF-β, p- STAT3, and t-STAT3 were increased in the miR-320 OE group compared with the model group, whereas PTEN was reduced in the miR-320 OE group. (Figure 5B, *P* < 0.05) These results suggested that overexpression of miR-320 might heighten TGF-β, IL-6, p-STAT3 and suppress PTEN in cardiac hypertrophy and fibrosis.

### PTEN was involved in the miR-320 mimics-induced development of hypertrophy and fibrosis *in vitro*

**Figure 6:**
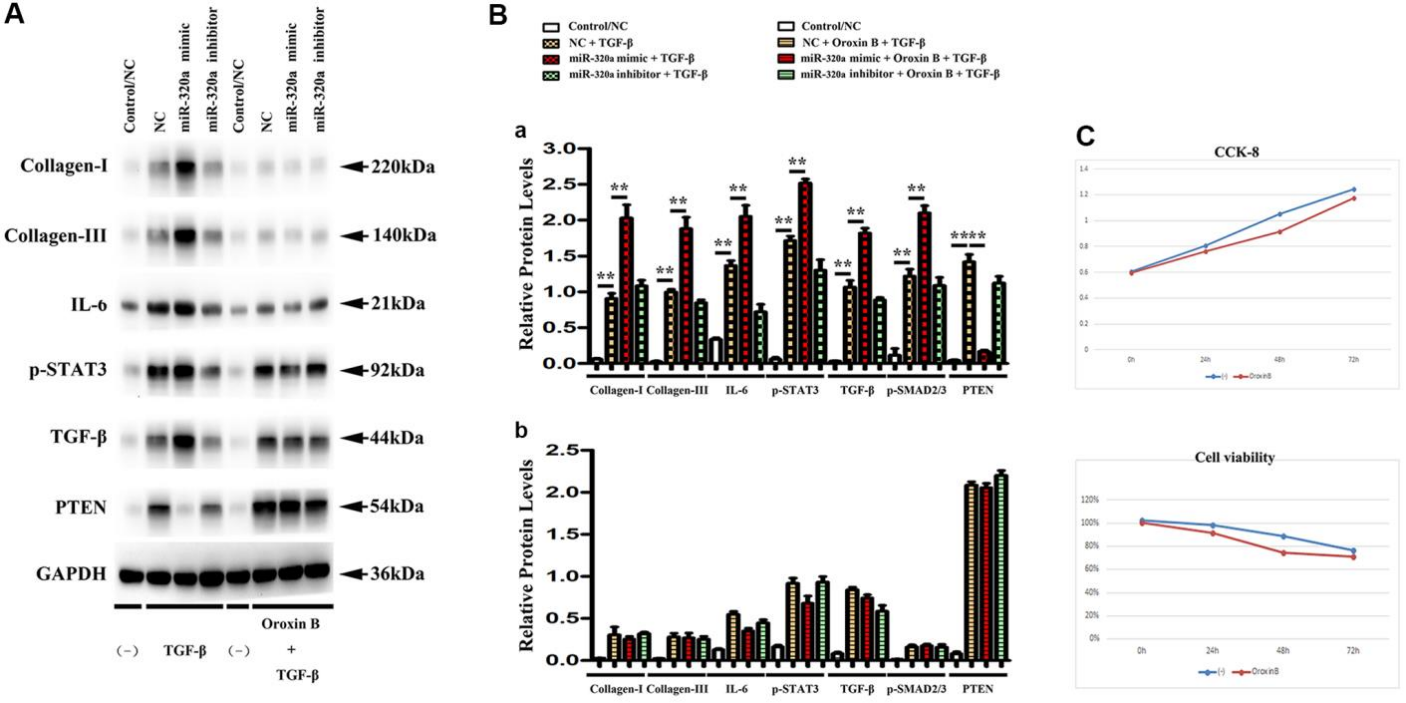
**PTEN was involved in miR-320 mimics-induced cardiac fibrosis.** (**A**) Representative Western blots in fibroblasts with or without orexin B injection; (**B**) The quantitative analysis of TGF-β, IL-6, p-STAT3, and PTEN with or without orexin B injection; *P* < 0.05. (**C**) The proliferation of fibroblasts treated with miR-320 mimics, inhibitor or negative control (NC) was measured by CCK8 assay.

It is a consensus that cardiac fibroblasts are crucially involved in the pathophysiology of cardiac hypertrophy and fibrosis [21]. To investigate whether PTEN was involved in miR-320 mimics-induced cardiac hypertrophy and fibrosis, orexin B, an agonist that up-regulates PTEN expression, was injected into the culture medium of fibroblasts transfected with miR-320 mimics. In western blots, we found that the decrease in PTEN was reversed by injecting orexin B in fibroblasts transfected with miR-320 mimics. (Figure 6A) Moreover, the enhanced type I and type III collagen, IL6, p-STAT3, and TGF-β were all reversed by orexin B injection in fibroblasts transfected with miR-320 mimic. (Figure 6A) The quantitative analysis of relative protein levels further confirmed that orexin B significantly reduced increased levels of type I and III collagen, IL6, p-STAT3, and TGF-β in fibroblasts transfected with miR-320 mimic (Figure 6B, *P* < 0.05), suggesting that PTEN was involved in the miR-320 mimics-induced development of cardiac hypertrophy and fibrosis. The viability of cardiac fibroblasts was investigated by CCK8 assay. The results revealed that orexin B administration had no effect on the migration of fibroblasts (Figure 6C).

### STAT3 was involved in miR-320 mimics-induced development of cardiac hypertrophy and fibrosis *in vitro*

**Figure 7:**
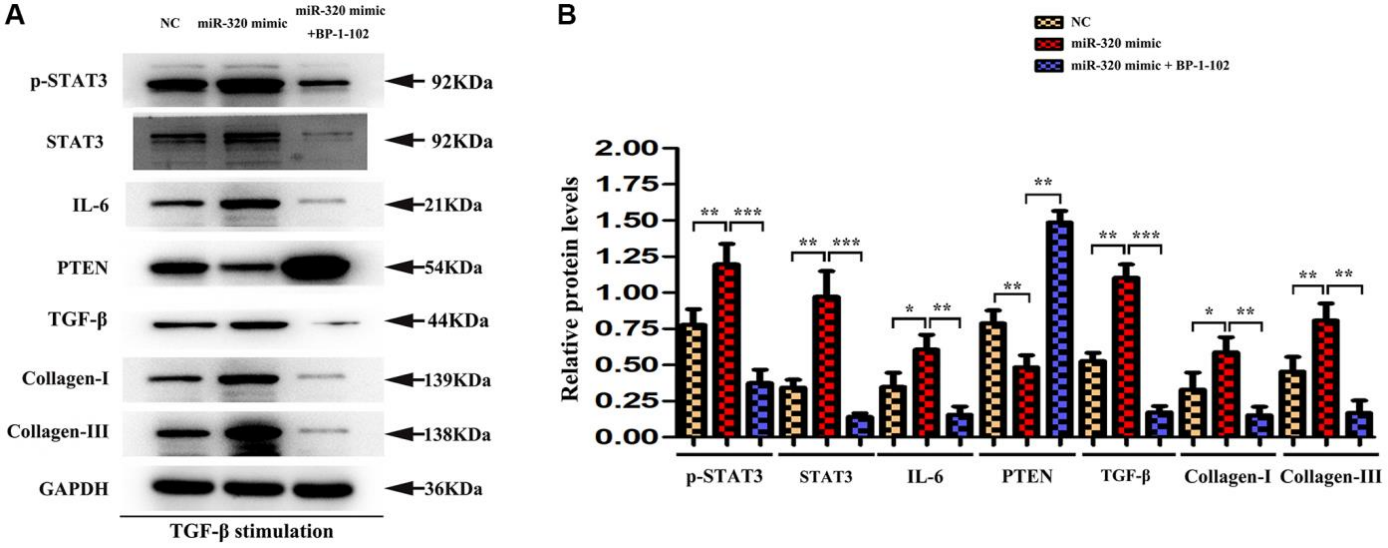
**STAT3 was involved in miR-320 mimics-induced development of cardiac hypertrophy and fibrosis.** (**A**) Western blots revealed that BP-1-102 significantly reversed the up-regulated STAT3, p-STAT3, type I and type III collagen, IL6, TGF-β, and down-regulated PTEN. (**B**) The quantitative analysis illustrated that inhibitor BP-1-102 exposure blunted the up-regulated type I and III collagen, IL6, TGF-β, p-STAT3, and STAT3 expression and enhanced the down-regulated PTEN expression in fibroblasts transfected with miR-320 mimic by BP-1-102. *P* < 0.05.

To investigate whether STAT3 signaling was involved in the overexpression of miR-320-induced cardiac hypertrophy and fibrosis, BP-1-102, an inhibitor of STAT3, was added into the culture medium of cardiac fibroblasts transfected with miR-320 mimic and inhibitor. In Western blots, we found the expression of p-STAT3 were significantly decreased in fibroblasts transfected with miR-320 mimic by the stimulation with BP-1-102. (Figure 7) Furthermore, the up-regulated type I and type III collagen, IL6, TGF-β, and down-regulated PTEN were significantly reversed by BP-1-102. (Figure 7) Our quantitative analysis illustrated that inhibitor BP-1-102 exposure blunted the up-regulated type I and III collagen, IL6, TGF-β, p-STAT3, and STAT3 expression and enhanced the down-regulated PTEN expression in fibroblasts transfected with miR-320 mimic by BP-1-102. (Figure 7, *P* < 0.05). The results implied that STAT3 was involved in miR-320 mimics-induced cardiac hypertrophy and fibrosis.

## DISCUSSION

In the current study, we proposed some novel findings. We found that miR-320 expression was decreased with the development of cardiac fibrosis. Besides, miR-320 was negatively correlated with KLF9 protein. In contrast, miR-320 was positively associated with JAK/STAT3 signaling pathway. Our results further confirmed that miR-320 exacerbated cardiac fibrosis through activation of the IL6/STAT3 axis.

Primarily, the present study exposed that miR-320 had low expression levels in cardiac fibrosis tissues from the data in GSE104150. Furthermore, STAT3 was significantly highly expressed in cardiomyocyte lines from the DEGs based on the GSE124176 dataset. Gene set enrichment analysis (GSEA) revealed that miR-320 activation and STAT3 signaling pathways were positively connected. The relative expression levels of serum miR-320 were significantly elevated in patients with cardiac fibrosis, suggesting that miR-320 played a role in cardiac fibrosis. We exposed that miR-320 regulated the IL6/STAT3 signaling pathway in cardiac fibrosis by miRNA array analysis. Based on these data, we proposed to explore the influence of miR-320 on cardiac fibrosis.

In addition, miR-320 (overexpression of miR-320) synthetic mimics and model mice were subjected to pressure overload by transverse aortic constriction (TAC) operation to generate cardiac fibrosis mice models. After the TAC operation, miR-320 mimics exhibited severe left ventricle dilation and regional contractile dysfunction. Cardiac dysfunction arose after TAC and was worse in miR-320 mimics, suggesting our cardiac fibrosis and hypertrophy models were established successfully, and overexpression of miR-320 might induce worse left ventricular (LV) hypertrophy and cardiac function damage. Masson staining exposed severe myocardial interstitial, and perivascular fibrosis in miR-320 mimics hearts after TAC operation. Furthermore, the quantitative analysis of fibrosis area and heart/weight ratio further confirmed that cardiac fibroblasts were significantly enhanced in the miR-320 mimics heart compared with the model heart (*P* < 0.05), suggesting overexpression of miR-320 accelerated the cardiac fibrosis. Besides, immunofluorescence staining, and western blot illustrated type I and type III collagen synthesis were significantly increased in the left ventricular tissues obtained from miR-320 mimics compared with model mice, suggesting overexpression of miR-320 accelerated the disruption of cardiac function and cardiac remodeling by increasing the collagenous fibers synthesis.

Next, we focused on IL6 and STAT3 signaling pathways through a series of *in vivo* and *in vitro* experiments. The western blot exposed that protein expression levels of IL6, p-STAT3, and TGF-β were enhanced in miR-320 mimics, suggesting that overexpression of miR-320 induced the increase in STAT3 and IL6 activation *in vivo*. By contraries, the expression level of PTEN was reduced in miR-320 mimics. These results are in line with the notion that PTEN negatively correlates to STAT3-signaling activation [15]. *In vitro* experiment, orexin B, an agonist that could up-regulate the expression of PTEN, was injected into the culture medium of cardiac fibroblasts transfected with miR-320 mimic. We found that down-regulated PTEN was reversed by orexin B, and the enhanced type I and type III collagen, TGF-β, IL6, p-STAT3, STAT3 were also reversed by orexin B, suggesting PTEN was involved in the overexpression of miR-320-induced cardiac hypertrophy and fibrosis *in vitro*. The cell-counting kit-8 assay revealed that the proliferation ability of cardiac fibroblasts with anti-IL-6-neutralizing antibody (IL6 inhibitor) was attenuated in a time-dependent manner. By contraries, the proliferation ability of cardiac fibroblasts without IL-6-neutralizing antibody increased in a time-dependent manner. Additionally, orexin B administration had no effect on the migration of fibroblasts. These results implied that IL6 was also involved in the overexpression of miR-320-induced cardiac fibrosis development *in vitro*. What is more, BP-1-102, an inhibitor of STAT3, was injected into the culture medium of cardiac fibroblasts transfected with miR-320 mimic to investigate whether STAT3 signaling was involved in the overexpression of miR-320-induced development of cardiac hypertrophy and fibrosis. As shown in Figure 7, the expression of STAT3 and p-STAT3 were decreased in miR-320 mimic by the stimulation with BP-1-102. Furthermore, the up-regulated type I and type III collagen, IL6, TGF-β, and down-regulated PTEN were also reversed by inhibitor BP-1-102 in miR-320 mimic, suggesting STAT3 was involved in the overexpression of miR-320-induced cardiac hypertrophy and fibrosis *in vitro*. Here we report our surprising finding that IL6/STAT3 signaling pathways were involved in the overexpression of miR-320 induced cardiac fibrosis and hypertrophy.

In conclusion, the present study shows that overexpression of miR-320 accelerates cardiac hypertrophy and fibrosis induced by pressure overload by activating the IL6/STAT3 signaling pathway, indicating an excellent possibility that miR-320 may provide new ideas for the treatment of cardiac fibrosis and heart failure.
