# Correction for: miR-320 accelerates chronic heart failure with cardiac fibrosis through activation of the IL6/STAT3 axis

**DOI:** 10.18632/aging.205256

**Published:** 2023-10-30

**Authors:** Fang Li, Shan-Shan Li, Hui Chen, Jian-Zhi Zhao, Jie Hao, Jin-Ming Liu, Xiu-Guang Zu, Wei Cui

**Affiliations:** 1 Third Division, Department of Cardiology, The Second Hospital of Hebei Medical University, Shijiazhuang, Hebei 050011, PR China; 2 Department of Biochemistry and Molecular Biology, The Hebei Medical University, Shijiazhuang, Hebei 050011, PR China; 3 Department of Cardiology, The Second Hospital of Hebei Medical University and Hebei Institute of Cardiovascular Research, Shijiazhuang, Hebei 050011, PR China

**Keywords:** miR-320, chronic heart failure, cardiac fibrosis, IL6/STAT3/PTEN axis

**This article has been corrected:** The authors found an error in **Figure 7A**: the Western blot band for Collagen-I was mistakenly also placed at the position for IL-6. The authors prepared a new **Figure 7** using images from the original experiments and recalculated the relative densities of these proteins. This correction does not impact the conclusions of the paper.

New **Figure 7** is presented below.

**Figure 7:**
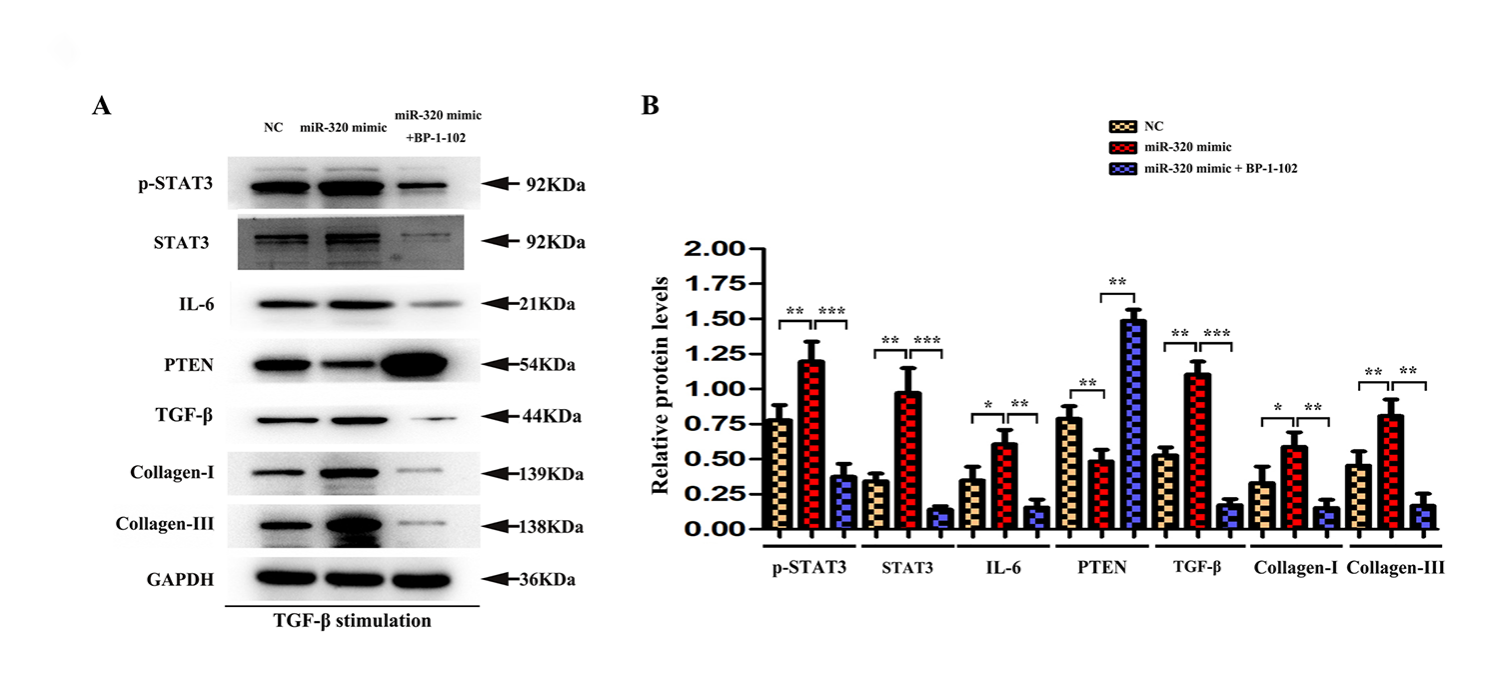
**STAT3 was involved in miR-320 mimics-induced development of cardiac hypertrophy and fibrosis.** (**A**) Western blots revealed that BP-1-102 significantly reversed the up-regulated STAT3, p-STAT3, type I and type III collagen, IL6, TGF-β, and down-regulated PTEN. (**B**) The quantitative analysis illustrated that inhibitor BP-1-102 exposure blunted the up-regulated type I and III collagen, IL6, TGF-β, p-STAT3, and STAT3 expression and enhanced the down-regulated PTEN expression in fibroblasts transfected with miR-320 mimic by BP-1-102. *P* < 0.05.

